# Ongoing Response in BRAF V600E-Mutant Melanoma After Cessation of Intermittent Vemurafenib Therapy: A Case Report

**DOI:** 10.1007/s11523-015-0410-9

**Published:** 2016-02-09

**Authors:** Andrew J. Dooley, Avinash Gupta, Mark R. Middleton

**Affiliations:** 1 Department of Oncology, NIHR Biomedical Research Centre, Oxford Cancer and Haematology Centre, Churchill Hospital, Old Road, Headington, Oxford, OX3 7LE UK; 2 University of Oxford, John Radcliffe Hospital, Oxford, OX3 9DU UK

**Keywords:** Melanoma, Gefitinib, Metastatic Melanoma, Ipilimumab, Vemurafenib

## Abstract

The selective BRAF inhibitors vemurafenib and dabrafenib yield high response rates and improved overall survival in patients with BRAF V600E-mutant metastatic melanoma. Treatment traditionally continues until disease progression or the development of unacceptable toxicity. Acquired drug resistance and toxicity are key challenges with the use of these drugs. Resistance to vemurafenib usually develops within 6–8 months. Management of drug toxicity typically involves stopping vemurafenib until resolution, before restarting at a lower dose, or permanently ceasing vemurafenib therapy. We have recently considered whether intermittent dosing could be used as an alternative to dose reduction/termination in the management of vemurafenib toxicity. One patient treated with intermittent vemurafenib was an 89-year-old woman with metastatic melanoma, who initially showed a good response to continuous dosing. Recurrent toxicity meant that the continuous vemurafenib dosage was repeatedly ceased before restarting at a lower dose. Ten months after vemurafenib was first begun, an intermittent dosing regimen was introduced in an attempt to control toxicity. This continued for 2 months, before cessation due to continued unacceptable toxicity. A further 24 months later, the patient remains fit and well in complete clinical remission, with no recurrence of her previous melanoma and no new primary malignancies. To the best of our knowledge, a continued response after the cessation of selective BRAF inhibitors has never before been described in melanoma. Induction of an immune response and/or epigenetic changes could explain continued disease response after cessation of vemurafenib therapy. Care should be taken when extrapolating the findings from the continued response after vemurafenib cessation to other tumour types. We recommend the collection and analysis of data to investigate the clinical responses seen after cessation of vemurafenib due to intolerable toxicities, which could help further explain vemurafenib’s mechanism of action.
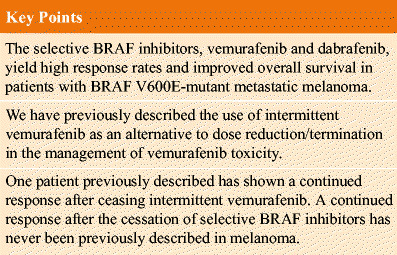

## Background

The v-raf murine sarcoma viral oncogene homolog B1 (BRAF) gene is mutated in 40–60 % of melanomas, the most common being the V600E mutation, which leads to over-activation of the mitogen-activated protein kinase (MAPK) pathway [1, 2]. The selective BRAF inhibitors vemurafenib and dabrafenib yield high response rates and improved overall survival in patients with BRAF V600E-mutant metastatic melanoma [3, 4]. However, acquired drug resistance and drug toxicity are key challenges with the use of these drugs. Resistance to vemurafenib usually develops within 6–8 months [5]. In the BRAF Inhibitor in Melanoma-3 (BRIM-3) trial, a phase 3 randomised open-label study comparing vemurafenib with dacarbazine in BRAF V600E- and BRAF V600K-mutant melanoma, 38 % of patients receiving vemurafenib required dose modifications because of toxicity [3]. Management of toxicity typically involves stopping vemurafenib until resolution, before restarting at a lower dose, or permanently ceasing vemurafenib therapy. In an extended follow-up of BRIM-3, treatment was discontinued because of adverse events in 24 patients receiving vemurafenib and six patients receiving dacarbazine. No data were presented on the outcome of these patients after cessation of therapy due to unacceptable toxicities [6]. Although disease progression has traditionally been seen as the point at which vemurafenib therapy should cease, recent research has suggested that treatment beyond progression might be beneficial [7]. Acquired resistance to targeted therapies is also a challenge in the treatment of non-small cell lung cancer (NSCLC). Epidermal growth factor receptor (EGFR) inhibitors such as gefitinib, erlotinib and axitinib are standard first-line treatments for patients with NSCLC with activating *EGFR* mutations [8]. Unfortunately, most patients eventually acquire resistance, and management options after progression on EGFR TKIs have yet to be defined [9, 10]. The recent IMPRESS trial investigated whether targeted therapies should be continued after progression. The study concluded that continuation of gefitinib after radiological disease progression on first-line gefitinib did not prolong progression-free survival in patients who received platinum-based doublet chemotherapy as a subsequent line of treatment [8].

We recently presented a case series in which patients with intolerable toxicities on a continuous dosing regimen of vemurafenib were treated instead with an intermittent regimen. Our experience showed that intermittent dosing could successfully manage vemurafenib toxicities where continuous dosing at a reduced dose did not, while maintaining or achieving melanoma shrinkage. We therefore recommended that intermittent dosing should be considered as an alternative to dose reduction/termination in the management of vemurafenib toxicity [11]. While we could present no evidence that intermittent therapy is more effective than continuous therapy, or that it can prevent the appearance of drug-resistant disease, this is due to be investigated in clinical trials currently in set-up. In animal models, however, there is evidence which suggests that intermittent dosing of vemurafenib can forestall the emergence of resistance [12]. This study of an animal model of melanoma did not investigate whether intermittent dosing continued to exert an effect after cessation.

## The Case

We previously reported the case of an 88-year-old woman diagnosed with metastatic melanoma who showed a good response to vemurafenib despite several dose reductions due to toxicity [11].

She originally had a superficial spreading melanoma removed from her left leg in 2006 when she was 80 years old. The primary melanoma had a Breslow thickness of 1.5 mm and was non-ulcerated, with a mitotic index of 0.1–0.5 per mm^2^. No adjuvant therapy was given afterwards. A further melanoma was excised from the left leg in 2007 (again a superficial spreading melanoma, non-ulcerated, Breslow thickness 2.5 mm, mitotic count 3 per mm^2^). The patient continued on surveillance follow-up. She remained well until 2011, when she developed multiple rapidly growing and painful subcutaneous lesions along her left leg, confirmed as melanoma on biopsy. A CT scan in November 2011 showed two 11-mm nodules in the right lung, suggesting small-volume metastatic disease. Her melanoma was confirmed as containing the BRAF V600E mutation, and she was commenced on vemurafenib at the standard starting dose of 960 mg twice daily (BD) in January 2012, as part of an expanded access programme. A further CT scan just before starting treatment showed disease progression, with multiple small-volume pulmonary nodules and multiple enlarged left inguinal lymph nodes. Clinically, the disease in her left leg had also worsened. Pre-treatment LDH was elevated at 262 IU/L (normal range: 110–255).

The patient showed a rapid clinical response to therapy, with a reduction in the leg lesions within 4 weeks of starting treatment. However, she also experienced various toxicities, particularly joint pain and nausea. After 8 weeks of therapy, a CT scan showed the majority of pulmonary nodules and inguinal lymph nodes had either significantly shrunk or completely resolved. One nodule in the left lower lobe, however, had enlarged. A further CT scan after 16 weeks of therapy showed the left lower lobe pulmonary nodule remained unaltered with no new metastases. Clinically, the lesions in her left leg continued to shrink. However, by week 24 of therapy, she had lost 14 kg and developed grade 2 nausea and diarrhoea. Vemurafenib was ceased at this point. These toxicities quickly resolved, so therapy was restarted a week later at a lower dose of 720 mg BD. However, the patient again developed toxicity within less than 2 weeks. Treatment was again ceased for 1 week, before restarting at 480 mg BD. Treatment continued at this lower dose for 8 weeks, before a further 1-week cessation due to toxicities. This pattern continued, with toxicity settling whilst off treatment but quickly re-emerging on restarting therapy. So, in November 2012, 10 months after initially starting vemurafenib, with CT and clinical evidence of ongoing response, a decision was made to recommence vemurafenib on an intermittent regimen of 480 mg BD, 1 week on, 1 week off. As this could not be done within the expanded access programme, we applied for and received approval for this to be funded through the Cancer Drugs Fund. The lesions on the left leg continued to shrink on intermittent dosing, and 1 month later the leg was clear of all visible deposits. However, intermittent dosing was ceased in January 2013 because of grade 2 arthralgia in the patient’s hands. A CT scan performed 4 weeks after cessation of vemurafenib showed an ongoing response, with no evidence of any metastatic disease. The marker left inguinal lymph node remained unchanged in size. We planned to restart vemurafenib as and when the leg lesions recurred, but so far this has not occurred. As of February 2015, the patient is alive and clinically free of disease. She has not needed any other treatment for her metastatic melanoma. A summary of her clinical history is presented in Table 1.

**Table 1:** Chronological details of patient's treatment, disease state and toxicities

Date	Vemurafenib therapy	Clinical disease status	Radiological disease status	Key toxicities (graded according to CTCAE v4.0)
19/01/2012		Multiple rapidly growing and painful lesions in left leg	Multiple sub-cm lung nodules. Several enlarged left (L) inguinal LNs, largest 19 mm (SAD)	
26/01/2012	Vem started at 960 mg BD continuous	Leg lesions getting worse; nodules growing in size and bleeding	-	
23/02/2012	Week 4 Continued on Vem 960 mg BD continuous	Good response, with decrease in size of lesions and pain	-	Nausea G1, Joint pain G1, Diarrhoea G1
15/03/2012	Week 7 Continued on Vem 960 mg BD continuous	Ongoing response	Lung nodules largely resolved. One nodule L lower lobe bigger. Decrease in L inguinal LNs. Largest 9 mm (SAD)	Nausea G1, Joint pain G1, Diarrhoea G1
10/05/2012	Week 15 Continued on Vem 960 mg BD continuous	Ongoing response	L lower lobe nodule unchanged. No other lung nodules. Marker L inguinal LN 8 mm (SAD)	Nausea G1, Joint pain G1, Diarrhoea G1, Skin toxicity G1
12/07/2012	Week 24 Vem stopped for 1 week	Ongoing response		Nausea G2, Diarrhoea G2, Vomiting G1, Joint pain G1, Skin toxicity G1, Fatigue G1
19/07/2012	Week 25 Vem restarted at 720 mg BD continuous	Ongoing response		Nausea G1
30/7/2012	Week 27 Vem stopped for 1 week	Ongoing response		Nausea G1, Anorexia G1
06/08/2012	Week 28 Vem restarted at 480 mg BD continuous	Ongoing response		Improvement whilst off Vem. Return of previous toxicities on restarting
29/8/12	Week 31 Continued on Vem 480 mg BD continuous	Ongoing response	No significant lung nodules. Marker L inguinal LN 8 mm (SAD)	Joint pain G1, Skin toxicity G1, Fatigue G1
4/10/2012	Week 36 Vem stopped for 1 week	Complete response (no visible lesions on leg)		Joint pain G2, Fatigue G2, Skin toxicity G1
13/10/2012	Week 37 Vem restarted at 480 mg bd continuous	Ongoing complete response		Improvement whilst off Vem. Return of previous toxicities on restarting
16/11/2012	Week 43 Vem stopped for 1 week	Ongoing complete response		Nausea G1, Joint pain G1 Skin toxicity G1, Diarrhoea G1, Fatigue G1
23/11/2012	Week 44 Vem restarted at 480 mg BD intermittent	Ongoing complete response	No significant lung nodules. Marker left inguinal LN 7 mm (SAD)	Improvement whilst off Vem
10/1/2013	Week 50 Vem discontinued	Ongoing complete response		Joint pain G2, Fatigue G2
07/02/2013		Ongoing complete response	No significant lung nodules. Marker L inguinal LN 7 mm (SAD)	Joint pain G1
20/09/2013		Ongoing complete response		Resolution of all toxicity

Abbreviations: *BD* twice-daily dosing, *CTCAE* common toxicity criteria for adverse events, *LN* lymph node, *SAD* short axis dimension, *Vem* vemurafenib

## Discussion

To the best of our knowledge, a continued response after ceasing vemurafenib has not been previously described in metastatic melanoma. The melanoma showed a rapid response soon after starting vemurafenib on a continuous dosing regimen. This response continued on intermittent dosing, with the leg lesions continuing to shrink until they completely disappeared. We feared the disease would recur soon after vemurafenib was ceased. However, the complete response has continued for over 2 years after cessation of therapy. It is difficult to understand what role, if any, intermittent therapy played in this continued response, especially since such a rapid, although not universal, response to continuous dosing was seen. Although the patient is in complete clinical remission, this does not mean the melanoma has been completely eliminated. It is possible there are small areas of residual disease left in this patient, and recurrence could yet occur many years later. Since late recurrence of melanoma is not uncommon, no melanoma patient, including the one detailed in this report, can ever be considered cured [13].

### Continued Response in Non-Melanoma BRAF Mutated Cancer

A continued response after ceasing vemurafenib therapy has been described in a patient with BRAF V600E-mutant hairy cell leukaemia (HCL) [14]. Briefly, this patient was treated with multiple lines of conventional therapy, but none proved effective. The presence of a V600E mutation was demonstrated, and so the patient was started on vemurafenib. The patient showed a rapid response to treatment within days, with a reduction in splenomegaly and rise in platelet, haemoglobin, and leukocyte counts. By day 35, flow cytometry showed no evidence of HCL cells in peripheral blood. The patient was treated with vemurafenib for a total of 56 days; the decision to cease therapy was made at this point because of the excellent response to treatment and to avoid cutaneous toxicities. Despite discontinuation of vemurafenib, the patient remained in remission, with no evidence of minimal residual disease 6 months after stopping treatment. Analysis of trephine biopsies obtained before and during treatment demonstrated abolition of ERK phosphorylation in HCL cells within 6 days of starting vemurafenib, resulting in apoptosis of the leukemic cells [14]. These findings support the conclusions drawn by in vitro work performed by Tiacci et al., in which BRAF-mutated HCL cells were shown to be highly sensitive to BRAF inhibition [15].

In the BRAF-mutant HCL patient who showed a continued response after ceasing vemurafenib, the drug appeared to mediate its effect by inhibiting the MAPK pathway. This is the same pathway targeted by vemurafenib in BRAF-mutant melanoma [16]. However, the continued clinical responses seen in these two cases are not necessarily the same phenomena. In the HCL case, the rapid elimination of all detectable HCL cells suggests that vemurafenib is acting on a homogenous clonal population of BRAF-mutant HCL cells. In our patient with melanoma, the response in most lesions, but the increase and subsequent stabilisation in one pulmonary lesion, suggests activity against a more heterogenous tumour population.

Although it is likely that MAPK pathway inhibition is necessary for clinical effectiveness of vemurafenib in both melanoma and HCL, different biochemical pathways may be involved. The genetic basis of HCL is not fully understood, but pathways involving FLT3L and IL3 appear to play an important role in protecting HCL cells from apoptosis [17]. In melanoma, different pathways are involved, such as the CDKN2A/CDK4 system and pathways involving the extracellular matrix not found in HCL [18, 19]. In the HCL case described above, it appears that BRAF inhibition interrupted signalling through MEK and ERK, which led to apoptosis in all of the clonal HCL cells, resulting (in this one case) in the effective elimination of the entire tumour population [14]. This suggests that all the BRAF-mutant HCL cells were dependent on aberrant BRAF signalling for survival. In BRAF-mutant melanoma, targeting the MAPK pathway alone is not sufficient to induce apoptosis in the entire tumour population. Much work on resistance to BRAF inhibition in melanoma has been done, and activation of the parallel PI3k/AKT pathway appears to play an important role [5, 20]. Although current understanding of HCL genetics is incomplete, the BRAF V600E mutation is found in the vast majority of HCL cases, compared with the 40–60 % of melanomas that contain a range of BRAF mutations [1, 21, 22]. Melanoma is the most mutated tumour in oncology, suggesting the presence of numerous alternate pathways that could play a role in resistance development [23].

There is now good evidence that melanoma metastases can be genetically heterogeneous, with genetic differences found both between and within different melanoma metastases in a single patient [24]. It is likely that different metastases will show different characteristics, both in terms of their natural history and in their response to vemurafenib. In this case report, the majority of melanoma metastases were very sensitive to vemurafenib, suggesting that the drug acted on these lesions directly through inhibition of ERK phosphorylation and subsequent apoptosis of cancer cells, as with the HCL case. However, the fact that one pulmonary lesion initially grew suggests it had less dependence on the MAPK pathway. The subsequent growth arrest with continued vemurafenib treatment suggests that the drug eventually worked via a different mechanism (possibly an immune-mediated response).

### Possible Explanations for the Continued Response

There are several possible explanations as to why the disease did not progress after cessation of treatment in the case of our patient. Firstly, the natural history of the disease needs to be understood; it could be that this patient had an indolent form of melanoma. Our patient is female; there is a much more favourable long-term survival for women after development of distant metastases [25]. However, we feel that this is an unlikely explanation for this patient, given that prior to starting vemurafenib, the melanoma was growing rapidly, especially the leg lesions.

Other potential mechanisms by which vemurafenib could produce a lasting response, even after cessation of treatment, could include the induction of an immune response and/or epigenetic mechanisms.

### Epigenetic Modification

It is increasingly understood that epigenetics plays a role in the pathogenesis of cancer, and several epigenetic modifiers are in development, where the drug targets enzymes involved in epigenetic control of gene expression. These targets include DNA methyltransferases and acetyltransferases. Epigenetic modifiers such as azacitidine (a DNA methyltransferase) and vorinostat (a histone deacetylase) are now approved cancer therapies [26]. Epigenetic modification of gene expression can result in the drug continuing to exert its effect long after its administration has been ceased [27]. It is plausible that BRAF inhibition could induce epigenetic changes, leading to long-term effects which last beyond drug treatment. Given that acquired resistance to BRAF inhibitors in melanoma is likely due to upregulation of alternative pathways, it is plausible that epigenetic regulation plays a role in acquired resistance [5]. In our experience, after stopping continuous dosing of vemurafenib, there is often rapid progression of disease. Here, the fact that this patient was treated with intermittent therapy could be an important factor in the ongoing complete response after ceasing therapy, since it might have changed the epigenetic profile of the melanoma in a beneficial way that could not be achieved with continuous dosing. Work in animal studies has shown that intermittent therapy can prevent acquired resistance [12]. Further research investigating whether response to treatment continues after ceasing intermittent vemurafenib and how intermittent treatment affects the epigenetic profile of tumours would be helpful.

### Immune Induction

It is now clear that the immune system plays an important role in melanoma. Partial regression of primary cutaneous melanomas is a common event, though complete regression is rare [28]. Spontaneous regression of metastatic melanoma is very rare [29]. There are many potential mechanisms by which this might occur, but the most likely is the activation of the immune system to produce a greater than normal response. Many immunomodulating drugs have now been shown as effective treatments in melanoma. Interferon-α2 and interleukin 2 are approved for use in an adjuvant setting in selected cases [30, 31]. Ipilimumab is an anti-CTLA-4 monoclonal antibody which augments T-cell activation. Ipilimumab was the first drug to improve overall survival in the metastatic setting [32]. IMCgp100 is a novel immunotherapy currently in phase 1 trials [33]. Perhaps most significant has been the development of PD-1 inhibitors. Nivolumab and pembrolizumab are both PD-1 inhibitors approved for the treatment of melanoma in the US and in Europe [34–36].

Recently, there has been interest in whether inhibiting the MAPK pathway up-regulates the immune response against melanoma metastases. If this is the case, it could provide a possible explanation for the ongoing complete response after vemurafenib therapy was ceased in our patient. One study, in which biopsies were taken from patients before and after starting vemurafenib therapy, showed that BRAF inhibition was associated with increased melanoma antigen expression, increased CD8+ T-cell infiltrate and decreased immunosuppressive cytokines in tumours of patients with metastatic melanoma. When patients progressed, biopsies were again taken, showing that melanoma antigen expression and CD8+ T-cell infiltrate were decreased at the time of progression [37]. These findings would be consistent with a model where decreased immune response allows the tumour to escape immune surveillance and therefore progress. Animal studies have shown that host immunity can contribute to the anti-tumour activity of BRAF inhibition, with CCR2 suggested as an important participant [38]. It would be of interest to determine whether intermittent vemurafenib is associated with increased markers of an immune response compared with continuous vemurafenib. It would also be of interest to assess whether concurrent treatment with immunosuppressants, such as steroids, affects response to therapy with vemurafenib. Studies investigating the combination of BRAF inhibitors and immunotherapy in the treatment of metastatic melanoma are ongoing [38, 39]. Combined BRAF and MEK inhibition has now been found to be more effective that BRAF inhibition alone [40]. The effect of this combination on the immune system warrants further investigation.

It is worth noting that in our case, the patient suffered numerous toxicities which are possibly immune-related, particularly arthralgia. These continued despite dose reductions, and for some time after vemurafenib cessation. This may suggest that immune induction played a greater role in this patient than is typical with vemurafenib. That the response to therapy was rapid does not necessarily exclude an underlying immune component. Recent clinical trials combining ipilimumab and nivolumab in advanced melanoma have shown that over 80 % reduction in target lesion size within 6–12 weeks is not uncommon [41].

## Summary

In summary, over 24 months after ceasing intermittent vemurafenib therapy due to unacceptable toxicities, the patient described in this case remains well, with continued clinical complete response. To the best of our knowledge, a continued response of metastatic melanoma to vemurafenib after ceasing treatment has never before been described. Understanding how this occurred could provide greater insight into how vemurafenib exerts its effect in BRAF-mutant metastatic melanoma. We recommend follow-up of other patients who have had to cease vemurafenib due to unacceptable toxicities, in order to see how common the occurrence of continued response is after cessation of vemurafenib therapy, and whether the toxicity experienced can provide clues as to other mechanisms of action of this drug, beyond simple inhibition of the MAPK pathway.
